# Clinical Characteristics of *Nocardia* Infection in Patients with Rheumatic Diseases

**DOI:** 10.1155/2013/818654

**Published:** 2013-09-19

**Authors:** Mieko Yamagata, Koichi Hirose, Kei Ikeda, Hiroshi Nakajima

**Affiliations:** Department of Allergy and Clinical Immunology, Chiba University Hospital, 1-8-1 Inohana, Chiba, Chiba City 260-8677, Japan

## Abstract

Although *Nocardiosis* has considerable recurrence and mortality rates, characteristics and risk factors of *Nocardia* infection have not been assessed in patients with rheumatic diseases. Here, we examined the characteristics and risk factors of *Nocardia* infection in rheumatic disease patients in our hospital. Ten rheumatic disease patients who developed *Nocardia* infection were identified by retrospectively reviewing the medical records. Possible predisposing factors for *Nocardia* infection were high-dose glucocorticoid treatment, concomitant use of immunosuppressants, preexisting pulmonary diseases, and diabetes mellitus. All patients had pulmonary *Nocardiosis*, and six of them had disseminated *Nocardiosis* when their pulmonary lesions were identified.

## 1. Introduction

*Nocardia* species are ubiquitous environmental microorganisms which belong to a diverse group of bacteria known as aerobic actinomycetes [1]. More than 50 species of *Nocardia* have been identified, and at least 30 species of them have been reported to possess clinical significance [1]. The majority of *Nocardia* infections are caused by inhalation, which develops lung lesions called pulmonary *Nocardiosis*, while some infections are caused by traumatic percutaneous inoculation [2]. *Nocardia* species can spread from these primary infection sites to various organs hematogenously and develop a pathogenic condition called disseminated *Nocardiosis*. 

*Nocardia* infection mainly occurs in immunocompromised hosts, including patients with *Human Immunodeficiency Virus *(HIV) infection, those who underwent organ transplantation, and those who received long-term corticosteroid therapy [3]. Although the incidence of *Nocardia* infection is low, its early detection and treatment in patients at high risk are clinically important due to its high mortality rate [4, 5]. Therefore, it is desired to identify the risk factors and clinical characteristics of *Nocardia* infection in each clinical cohort of immunocompromised hosts. In patients who are organ transplantation recipients or are infected with HIV, administration of high-dose corticosteroids, a history of *Cytomegalovirus* (CMV) infection, and low CD4^+^ T-cell counts in peripheral blood have been reported as risk factors for* Nocardia* infection [6, 7]. Although case reports of *Nocardia* infection in patients with rheumatic diseases underscore its importance [8–10], the risk factors for *Nocardia* infection in patients with rheumatic diseases have not been assessed yet. In this study, we retrospectively reviewed the medical records of our hospital and assessed the risk factors, clinical features, and microbial characteristics of *Nocardia* infection in patients with rheumatic diseases.

## 2. Methods

### 2.1. Patients

Rheumatic disease patients who developed culture-proven *Nocardia *infection from January 1995 to July 2012 were retrospectively identified by reviewing medical records in the Department of Allergy and Clinical Immunology, Chiba University Hospital, and clinical information was collected from the records. Disseminated* Nocardia* infection was defined as involvement of 2 or more organs. 

### 2.2. Microbiology

*Nocardia* species were identified based on colonial and microscopic morphology and on the demonstration of partial acid-fast staining at the Microbiology Department in Chiba University Hospital.

## 3. Results

### 3.1. Clinical Features of Rheumatic Disease Patients Who Were Diagnosed with *Nocardia* Infection

 The demographics and characteristics of 10 rheumatic disease patients who were diagnosed with *Nocardia* infection are shown in Table 1. The underlying rheumatic diseases of the patients were as follows: microscopic polyangiitis (*n* = 3), systemic lupus erythematosus (SLE) (*n* = 2), Behçet's disease (*n* = 1), Sjögren's syndrome (*n* = 1), granulomatosis with polyangiitis (*n* = 1), adult-onset Still's disease (*n* = 1), and rheumatoid arthritis (RA) with vasculitis (*n* = 1). The mean time to develop *Nocardia* infection after the diagnosis of rheumatic diseases was more than 7 years, and 4 patients developed *Nocardia* infection more than 10 years after the onset of rheumatic diseases (Table 1). 

 The mean glucocorticoid dose at the onset of *Nocardia* infection was 19.7 mg (prednisolone equivalent)/day. Five patients were also receiving other immunosuppressants: azathioprine (*n* = 3), cyclosporine (*n* = 1), and intravenous administration of cyclophosphamide (*n* = 1) (Table 1). Although the association of anti-TNF therapy with *Nocardiosis* has been suggested [11, 12], none of our patients with *Nocardia* infection were receiving anti-TNF therapy. One patient developed *Nocardia* infection even though the patient was taking Trimethoprim-sulfamethoxazole (TMP-SMZ), the most commonly used antibiotics against *Nocardia*, for prophylaxis against *pneumocystis jiroveci* (Table 1).

Eight out of the 10 patients had diabetes mellitus, and 4 patients were poorly controlled (glycated hemoglobin [HbA1c] < 7.0%) (Table 2). Seven out of the 10 patients had pulmonary diseases including pulmonary lesions induced by underlying rheumatic diseases, a history of pulmonary tuberculosis, and pulmonary *aspergillosis* (Table 2). In contrast to the previous reports suggesting the association between *Nocardia* infection and lymphocytopenia [13, 14], white blood cell (WBC) counts and lymphocyte counts in peripheral blood in our patients were within normal limits (Table 2). In addition, no patients had severe hypogammaglobulinemia or hypoalbuminemia. These results suggest that treatment with high-dose glucocorticoid, concurrent use of immunosuppressants, and preexisting pulmonary diseases are associated with the development of *Nocardia* infection in patents with rheumatic diseases, which is consistent with the previous report on the patients with organ transplantation [6], and that the presence of diabetes mellitus further increases the risk of *Nocardia* infection in patients with rheumatic diseases. 

### 3.2. Characteristics of *Nocardia* Infection in Patients with Rheumatic Diseases

 The strains of *Nocardia* species isolated from the patients with rheumatic diseases are shown in Table 3. *N. farcinica* was the most common species in our patients (*n* = 5). All patients were diagnosed in outpatient settings and had pulmonary *Nocardiosis*. Importantly, intensive examination revealed that 6 out of the 10 patients had disseminated diseases (brain abscess (*n* = 3), multiple muscle abscess (*n* = 1), mediastinum abscess (*n* = 1), and subcutaneous abscess (*n* = 1)) when their lung lesions were detected (Table 3).

 It has been reported that carbapenem monotherapy or its combination with amikacin was well tolerated and effective for severe *Nocardiosis* [15]. Considering the immunocompromised state of our patients, 8 patients including 6 patients with disseminated diseases were treated with regimens containing intravenous administration of carbapenem antibiotics as an induction therapy (Table 3). All patients received the induction therapy for 8 to 12 weeks, followed by long-term treatment with oral TMP-SMZ as previously recommended [16]. Four patients failed to continue to take TMP-SMZ because of renal dysfunction or drug allergy and received substitution therapies such as long-term treatment with minocycline or quinolone antibacterial agents. Consequently, 9 patients including 6 patients with disseminated diseases achieved remission and had no recurrence of *Nocardia* infection (Table 3). One case of pulmonary *Nocardiosis* with severe interstitial pneumonitis associated with microscopic polyangiitis suffered from several recurrences in spite of the treatment with oral antibiotics (Table 3). Importantly, *N. farcinica* isolated from this patient gradually changed their susceptibility to antibiotics and finally acquired resistance to TMP-SMZ. 

## 4. Discussion

 Recent advances in immunosuppressive regimens against rheumatic diseases combined with antimicrobial prophylaxis strategies have led to significant alternation in the prevalence of opportunistic infections in patients with rheumatic diseases. Importantly, the introduction of antitumor necrosis factor (TNF)-*α* biologics has been shown to increase the incidence of granulomatous infections, including *Nocardiosis* [11, 12]. Furthermore, calcineurin inhibitors, a potential risk factor for *Nocardia* infection in organ transplant recipients [6], have become widely used for rheumatic diseases. To our knowledge, however, the risk factors and characteristics of *Nocardia* infection in patients with rheumatic diseases have not been reported. 

 In this study, we showed that the administration of high-dose glucocorticoid and concurrent use of immunosuppressants seem to be risk factors for *Nocardia* infection in patients with rheumatic diseases (Table 1) in consistency with the previous studies focusing on patients with organ transplantation or neoplastic diseases [6, 17]. In contrast to the previous reports which suggest the association of anti-TNF-*α* therapy with *Nocardiosis* [11, 12], we did not find any rheumatic disease patients who developed *Nocardia* infection under treatment with anti-TNF-*α* biologics in our small number of cases. 

 In addition to these drugs used for the treatment of underlying rheumatic diseases, our data suggest that diabetes mellitus and preexisting pulmonary diseases are risk factors for *Nocardia* infection in rheumatic disease patients. On the other hand, lymphocytopenia and CMV infection, which have been suggested to be associated with *Nocardia* infection in patients with organ transplantation or neoplastic diseases [6, 13, 14], were not identified in our patients with rheumatic diseases who developed *Nocardia* infection (Table 2) possibly due to the relatively low incidence of lymphocytopenia and CMV infection in these conditions [18, 19]. Taken together, these results suggest that the risk factors for development of *Nocardia* infection can be different in patients with rheumatic diseases compared to those with organ transplantation or neoplastic diseases presumably because of the differences in the preexisting immunological abnormalities and/or the therapy for underlying diseases. Further studies with larger sample size are needed to assess the detailed risk factors for *Nocardiosis* in patients with rheumatic diseases.

 Our results suggest that extrapulmonary lesions of* Nocardia* infection are frequently observed in patients with rheumatic diseases. We found that 6 out of the 10 patients had extrapulmonary abscesses when their pulmonary lesions were diagnosed (Table 3). Although it is well known that *Nocardia* species readily spread hematogenously, the proportion of disseminated *Nocardia* infection in our patients is higher than that in previous reports [6, 20]. At present, the reason for the high frequency of disseminated *Nocardia* infection in patients with rheumatic diseases is unknown. However, these results underscore the importance of the intensive examination for extrapulmonary lesions when the diagnosis of pulmonary *Nocardia* infection is made in patients with rheumatic diseases. 

## 5. Conclusion

Our results suggest that the predisposing factors for *Nocardiosis* in rheumatic disease patients are high-dose glucocorticoid therapy, concomitant use of immunosuppressants, preexisting pulmonary diseases, and diabetes mellitus. Our results also suggest that the intensive examination for extrapulmonary lesions is needed when the diagnosis of pulmonary *Nocardia* infection is made in patients with rheumatic diseases.

## Figures and Tables

**Table 1:** Demographics and characteristics of 10 rheumatic disease patients with* Nocardia* infection.

Case no.	Sex	Age	Underlying rheumatic disease	Treatment periods for rheumatic disease (years)	Doses of prednisolone (mg/day)	Other immunosuppressant	TMP-SMZ prophylaxis
1	M	70	Behçet's disease	>10	20		No
2	F	50	SLE	7	27.5	AZT	No
3	M	79	Sjögren's syndrome	1	20		No
4	F	77	Granulomatosis with polyangiitis	9	15		No
5	F	60	Adult-onset Still's disease	0.3	30	CyA	No
6	M	74	Microscopic polyangiitis	>10	4.5	AZT	Yes
7	F	67	SLE	>10	15		No
8	F	40	Microscopic polyangiitis	7	22.5	IVCY	No
9	M	69	Microscopic polyangiitis	5	25		No
10	M	62	RA with vasculitis	>10	17.5	AZT	No

SLE: systemic lupus erythematosus; RA: rheumatoid arthritis; AZT: azathioprine; CyA: cyclosporine; IVCY: intravenous administration of cyclophosphamide; TMP-SMZ: Trimethoprim-sulfamethoxazole.

**Table 2:** Comorbidities and clinical data of rheumatic disease patients with *Nocardia* infection.

Case no.	Pulmonary disease	Diabetes mellitus	HbA1c (%)	WBC (/*μ*L)	Lymphocyte (/*μ*L)	Alb (g/dL)	IgG (mg/dL)
1	Bronchial asthma	Yes	8.0	12400	409	2.7	705
2	No	No	4.5	8000	646	3.7	1111
3	Pulmonary tuberculosis	Yes	10.9	19600	585	3.6	1080
4	Interstitial pneumonitis	Yes	7.8	15100	1216	3.1	684
5	No	Yes	6.5	10400	1605	3.7	738
6	Pulmonary aspergillosis, Pulmonary tuberculosis	Yes	6.8	12200	1159	3.8	1451
7	No	Yes	6.7	9800	510	3.7	1083
8	No	No	6.2	13100	550	3.8	880
9	Pulmonary tuberculosis	Yes	9.6	17100	684	3.0	740
10	Interstitial pneumonitis	Yes	6.8	9100	536	3.7	789

WBC: white blood cell; Alb: albumin.

**Table 3:** Characteristics of *Nocardia* infection developed in rheumatic disease patients.

Case no.	*Nocardia* spp	Pulmonary nocardiosis	Extrapulmonary lesion	Initial therapy	Outcome
1	*N. asteroides *	Yes	Mediastinal abscess	IMP/CS + MINO + TMP-SMZ	Remission
2	N.D.	Yes	Brain abscess	PAPM/BP + CLDM + TMP-SMZ	Remission
3	*N. farcinica *	Yes	Iliopsoas abscess	MEPM	Remission
4	*N. nova *	Yes	Brain abscess	MEPM + TMP-SMZ	Remission
5	*N. farcinica *	Yes	No	MEPM + ABK	Remission
6	*N. farcinica *	Yes	No	MEPM	Recurrence
7	N.D.	Yes	Brain abscess	IMP/CS	Remission
8	N.D.	Yes	No	TMP-SMZ	Remission
9	*N. farcinica *	Yes	No	MEPM	Remission
10	*N. farcinica *	Yes	Subcutaneous abscess	TMP-SMZ	Remission

N.D.: not determined; IMP/CS: imipenem/cilastatin sodium; MINO: minomycin; TMP-SMZ: Trimethoprim-sulfamethoxazole.

PAPM/BP: panipenem-betamipron; CLDM: clindamycin; MEPM: meropenem; ABK: arbekacin.
